# Putting out the blaze: The neural mechanisms underlying sexual inhibition

**DOI:** 10.1371/journal.pone.0208809

**Published:** 2019-01-02

**Authors:** Geraldine Rodriguez-Nieto, Alexander T. Sack, Marieke Dewitte, Franziska Emmerling, Teresa Schuhmann

**Affiliations:** 1 Brain Stimulation and Cognition Lab, Department of Cognitive Neuroscience, Maastricht University, Maastricht, Netherlands; 2 Department of Clinical Psychological Science, Maastricht University, Maastricht, Netherlands; 3 School of Management, Technical University Munich, Munich, Germany; Victoria University of Wellington, NEW ZEALAND

## Abstract

The successful inhibition of sexual thoughts, desires, and behaviors represents an essential ability for adequate functioning in our daily life. Evidence derived from lesion studies indicates a link between sexual inhibition and the general ability for behavioral and cognitive control. This is further supported by the high comorbidity of sexual compulsivity with other inhibition-related disorders. Here, we aimed at investigating whether sexual and general inhibition recruit overlapping or distinct neural correlates in the brain. Furthermore, we investigated the specificity of two different kinds of sexual inhibition: inhibition of sexually driven motor responses and inhibition of sexual incoming information. To this end, 22 healthy participants underwent functional Magnetic Resonance Imaging (fMRI) while performing a task requiring general response inhibition (Go/No-go), as well as cognitive and motivational sexual inhibition (Negative Affective Priming and Approach-Avoidance task). Our within-subject within-session design enabled the direct statistical comparison between general and sexual inhibitory mechanisms. The general inhibition task recruited mainly prefrontal and insular regions, replicating previous findings. In contrast, the two types of sexual inhibition activated both common and distinct neural networks. Whereas cognitive sexual inhibition engaged the inferior frontal gyrus, the orbitofrontal cortex and the fusiform gyrus, motivational sexual inhibition was characterized by a hypoactivation in the anterolateral prefrontal cortex. Both types of sexual inhibition recruited the inferior frontal gyrus and the inferotemporal cortex. However, the activity of the inferior frontal gyrus did not correlate with behavioral inhibitory scores. These results support the hypothesis of inhibitory processing being an emergent property of a functional network.

## Introduction

Sexuality is one of the main driving forces underlying human behavior. Being essential for the individual’s ability to reproduce, sexuality is a deeply rooted and highly rewarding behavior. Nonetheless, the ability to inhibit sexual stimuli and control sexual behavior is of utter importance to convey to societal norms or to prevent harmful consequences. The failure to control sexual impulses and behavior may lead to a wide range of undesired consequences such as compulsive masturbation and pornography watching, undesired pregnancy, sexual transmitted diseases contagion, relationships problems, and sexual offending. It is, thus, plausible that specific sexual inhibitory mechanisms underlie the ability to control sexual responding.

The lack of control over sexual behavior has been studied in the context of compulsivity and addiction models. Hypersexual patients show typical addiction or compulsive symptoms, such as the interference with important occupational and social goals, and the repetitive and unsuccessful efforts in controlling their urges [1].

The high prevalence of substance abuse, impulse-control, and obsessive-compulsive spectrum disorders among hypersexual patients [2, 3] raises the question whether the control of sexual thoughts, urges, and behavior, may derive from a general inhibition ability that generalizes across many modalities. Indeed, some evidence suggests that general inhibition shows a certain degree of domain generality [4, 5]. A well-established and simple task to assess and quantify general response inhibition is the classic Go/No-go paradigm. Participants are required to respond to a frequent Go signal while inhibiting their response to a rare interleaved No-go signal. This Go/No-go paradigm has been used to study inhibition processes in drug addiction [6], aggression [7], cigarette craving [8], and sexual risk behavior [9–10].

Other studies have shown that the failure to respond to the Go stimulus (i.e. misses in the Go/No-go task) was associated with sexual arousability and sexual compulsivity [11, 12]. Moreover, convicted pedophilic sex offenders were found to react slower to Go stimuli (Go/No-go task) compared to control participants [13]. Although it is debatable whether the omission errors (misses) or slow reaction times in the Go/No-go task constitute an inhibition failure, in another study convicted pedophilic and non-pedophilic child molesters committed more errors (responding on No-go trials) than healthy controls and non-sex offenders [14].

The link between general and sexual inhibition is further supported by observations from lesion studies, in which frontal damage often leads to general disinhibition as well as sexual inhibition deficits [15]. However, there are also lesion studies demonstrating that damage to specific temporal regions, the septal region, and the pallidum, trigger specific sexual compulsive or deviant behaviors in some patients. Specifically, damage to temporal regions seem to cause a dramatic increase of sexual drive, but overall, non-frontal lesions seem unrelated to general inhibition impairments, as observed in frontal lesion cases [15]. The different symptoms and distinctive comorbidity of sexual disorders across patients suggest the existence of different sexual regulatory mechanisms that operate in addition to general inhibition networks in the brain. Although lesion studies provide valuable information on the link between inhibition and sexual behavior, an important limitation of these studies is that the damage is rarely focal. Epistemologically, even if a deficiency in general inhibitory mechanisms leads to a subsequent failure of inhibiting sexual behavior, that does not exclude the possibility that specific and/or additional sexual inhibitory mechanisms still exist. It is possible that different sexual manifestations (e.g. thoughts, behavior) engage different networks to be inhibited, which, depending on the process, could derive from a general and/or specific domain.

Previous neuroimaging studies have proposed the left lateral orbitofrontal cortex, the gyrus rectus, and the inferotemporal cortex to be tonic inhibitors of sexual arousal, showing distinct deactivation during the presentation of sexual stimuli [16]. In addition, the dorsolateral prefrontal cortex seems to play a regulatory role during and after the presentation of erotic films [17]. Probably the most direct evidence related to the neural correlates of sexual inhibition stems from a study in which participants were explicitly asked to inhibit any emotional reaction during the presentation of an erotic film. The superior frontal gyrus and anterior cingulate cortex (ACC) were active during the attempted inhibition of sexual responses [18].

On the other hand, neuroimaging studies investigating the neural correlates of general response inhibition using the Go/No-go paradigm, consistently describe mostly right-lateralized activation of the superior, middle, and inferior frontal gyri, the pre-supplementary motor area, the ACC, the inferior parietal lobe, the angular gyrus, basal ganglia, and anterior insula during the deliberate inhibition of a preponderant response [7,19–21]. Similarly, an erotic Go/No-go task engaged the dorsolateral prefrontal cortex, the anterior cingulate and the anterior insular cortices [10]. It is therefore plausible to assume that a common inhibitory network exists involving the dorsolateral prefrontal cortex, the anterior insula and the ACC. The possibility of an overlapping network for general and sexual inhibition has not been directly tested before. Moreover, the few neuroimaging studies that addressed sexual inhibition made mostly indirect inferences (i.e. based on the passive viewing of sexual stimuli). Hence, the question to what extent sexual inhibition comprises general inhibition mechanisms and which, if any, specific sexual inhibition networks are involved in different forms of sexual control is largely unanswered. For instance, it is possible that the mechanisms that allow the control of sexual thoughts are different from those that allow the control of sexually driven actions. To this regard, evidence comparing distinct aspects of sexual inhibition is missing.

Here, we aimed to characterize the neural substrates of sexual inhibition using functional Magnetic Resonance Imaging (fMRI) with two experimentally controlled paradigms assessing the motivational and cognitive aspects of sexual inhibition: Negative Affective Priming and Approach-Avoidance. We previously showed that the control of motivationally driven motor action tendencies (*motivational sexual inhibition* assessed by an Approach-Avoidance paradigm) behaviorally differs from the attentional control over sexual inputs (*cognitive sexual inhibition* assessed by a Negative Affective Priming paradigm) [22]. In addition, we included a classic response inhibition task (Go/No-go task) to directly investigate the potential overlap in neural correlates between sexual and general inhibition in a within-subject design.

## Method

### Participants

Twenty-four healthy male participants (18–34 years old) without neurological or psychiatric disorders took part in this study. One participant was excluded due to extensive head movements, and a second participant due to technical difficulties leading to an incomplete data set (final sample: N = 22, mean age = 24.77, SD = 4.76). Participants gave written informed consent and at the end of the session they received twenty euro in vouchers for their participation. The study was approved by the local Ethical Committee of the Faculty of Psychology and Neuroscience at Maastricht University.

### Procedure and instruments

Participants performed two sexual (Approach-Avoidance and Negative Affective Priming task) and one non-sexual (Go/No-go task) paradigm in an MR scanner. The order in which the two sexual tasks were presented was counterbalanced. The Go/No-go task was always presented between the sexual tasks to prevent habituation to sexual stimuli.

#### Approach-Avoidance task

**Fig 1:**
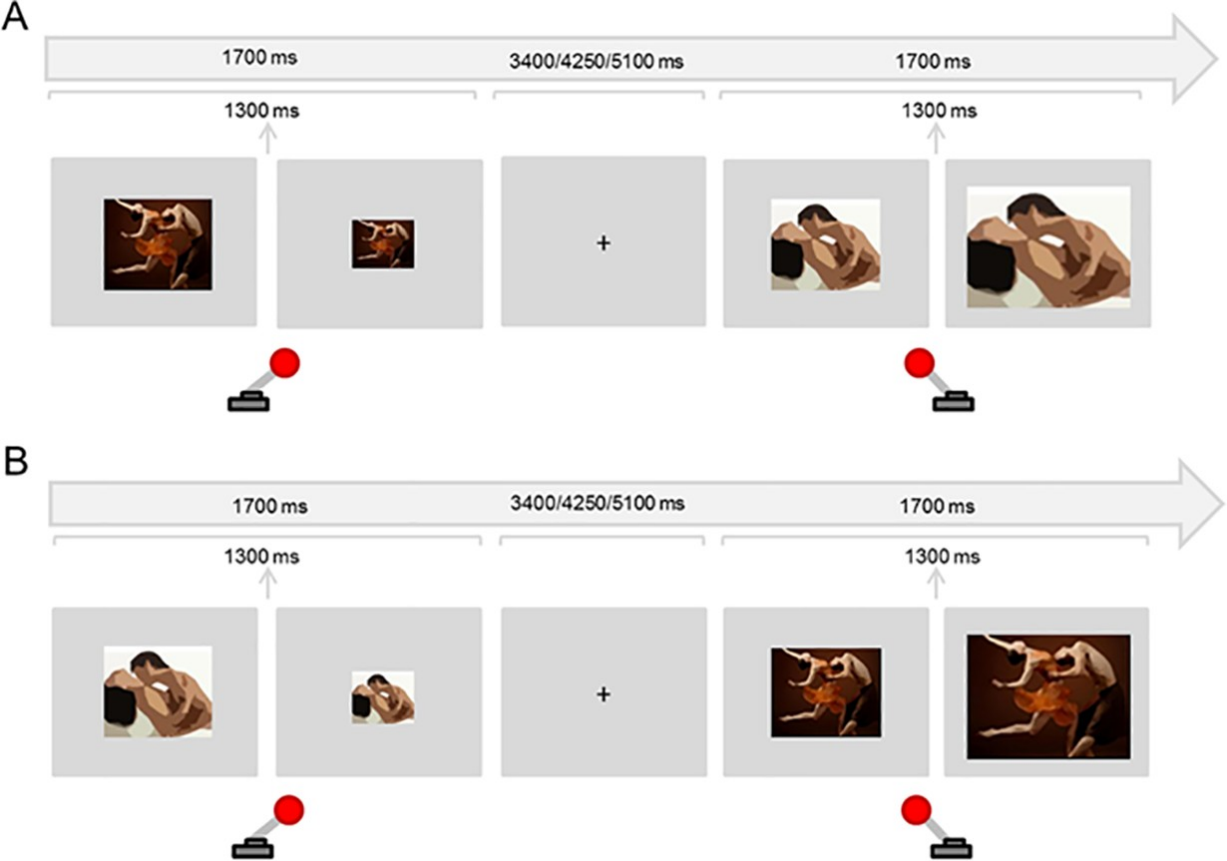
Motivational sexual inhibition—Approach-Avoidance task. A) Sex Approach block: Participants approached sexual images (pulling the joystick towards themselves increasing the image size) and avoided non-sexual photographs (pushing the joystick away from themselves decreasing the image size). B) Sex Avoid block: Participants avoided sexual images and approached non-sexual images.

This paradigm was selected to measure *motivational sexual inhibition* as it targets approach-avoidance tendencies towards stimuli with affective or neutral content (Fig 1). Previous adaptations using sexual stimuli have shown to be sensitive to gender differences [23], to be related to the amount of viewing time of erotic stimuli [24], and to predict pornography watching frequency [22].

There were four blocks of 48 randomized trials each. In two blocks, participants were instructed to approach the sexual stimuli (presented in 50% of the trials) and to avoid the non-sexual stimuli. For the other two blocks, participants were instructed to do the opposite. To approach, participants had to pull a joystick towards them. This action doubled the image size. To avoid, they had to push the joystick away from them, which in turn halved the image size. In every trial, the presentation of the stimulus lasted 1700 milliseconds. To avoid variability in timing across trials and participants, the resizing always occurred 1300 ms after stimulus onset. Between trials, a fixation cross was presented for 3400, 4250, or 5100 ms (Fig 1). The four blocks were counterbalanced.

Sexual stimuli were color photographs displaying sexual intercourse or oral sex between one woman and one man; 95% of these pictures were previously evaluated and validated in terms of valence and arousability [25]. The remaining pictures were selected from the internet. Non-sexual stimuli were color photographs of one woman and one man dancing [23]. The proportion of the exposition of the bodies (with special attention to the female body) with respect to the whole picture was comparable in both conditions. Images were displayed on a light gray background and the default size of the image was 337,9 X 272,5 pixels (horizontal orientation) in half of the blocks and 257,6 X 400 pixels (vertical orientation) in the other half. We calculated a Sex Approach-Avoid index, by subtracting the reaction times in Sex Approach blocks from reaction times in Sex Avoid blocks. A major Sex Approach-Avoid index indicated a stronger control over sexual motivation, by taking less time to avoid sexual stimuli and/or taking longer to approach them. The split-half reliability of this task was of .37 (p = .01).

#### Negative affective priming task

**Fig 2:**
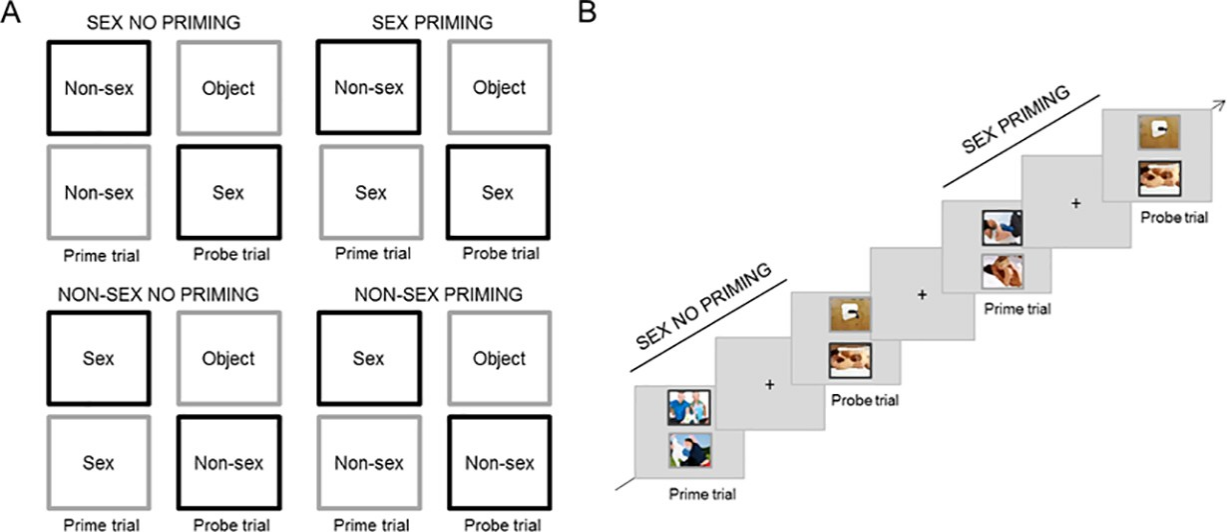
Cognitive sexual inhibition—Negative affective priming task. A) Priming conditions: the content of the distractor–gray frame- in the prime trial (Sex or Non-Sex) matched the content of the target–black frame- in the probe trial. No Priming conditions: the content of the distractor in the prime trial was different from the content of the target in the probe trial. B) Timeline: Example of a Sex No Priming and a Sex Priming trial sequence.

This paradigm (adapted from [23]) addressed *cognitive sexual inhibition* (Fig 2). Participants had to attend to one stimulus while ignoring a simultaneously presented distractor. In the next trial, the participant had to respond to the kind of stimulus that was previously ignored which causes a response delay (priming effect). The link between two successive trials was not obvious to the participant and, therefore, it can be argued that this task measures automatic inhibition (for an overview see [26]). The priming effect has shown to be larger for sexual than for neutral stimuli presumably due to a major implication of inhibition [22–23]. In addition, this task predicted the frequency of sexual thoughts in daily life [22].

The task consisted of four types of trial-sequences: a) Sex Priming, b) Sex No Priming, c) Non-Sex Priming, and d) Non-Sex No Priming. A trial-sequence contained a prime and a probe trial; during each, two pictures were presented simultaneously. The pictures were displayed one above the other; one surrounded by a black and the other by a gray frame. The instruction was to attend only to the picture with the black frame (target), therefore ignoring the one with the gray frame (distractor) and to indicate whether the target displayed sexual or non-sexual content. Participants responded by pressing a left or right button on a button box located below their right hand. During the priming trial-sequences, the content type of the distractor in the prime trial was the same as in the target picture of the probe trial. In the control trial-sequences (No Priming) the content type of the distractor in the primer trial and the target in the probe trial was different. The target of the probe trial could be sexual or non-sexual. Fig 2A provides an overview of the four different conditions.

The four types of trial-sequences were presented randomly in equal proportions throughout each of three blocks. Each block contained 32 trial-sequences and there were 24 trial-sequences in total for each condition. The prime and probe trials were presented for 1700 ms each. A fixation cross was displayed for 1700 ms between the prime trial and probe trial of the same sequence, and for 3400, 4250, or 5100 ms between different trial-sequences (Fig 2B). The sexual stimuli were pictures (320 x 260 pixels) different from those used in the AAT but with the same content characteristics. The non-sexual stimuli were photographs of one man and one woman exercising together. The neutral stimuli (distractors in the probe trials) were pictures of neutral objects (e.g. pencil case). Pictures were displayed on a light gray background and the picture frames were three pixels in width. 85% of photographs were selected from previously used data sets [23, 25], and the remaining were selected from the internet. A main sexual priming score was calculated by subtracting the sexual priming index (Sex Priming RT–Sex No Priming RT) minus the non-sexual priming index (Non-Sex Priming RT–Non-Sex No Priming RT). A higher index indicated a stronger sexual priming effect and thus, a stronger sexual inhibition. The split-half reliability of this task was of .31 (p = .21). The low reliability of the sexual tasks was possibly due to the large variability across stimuli. In spite of the reliability costs, such variability is desired as it allows generalization and ensures sensitivity. Because the images remain constant across participants and we compared the reaction times on the experimental versus control trials for the exact same images, the calculated indices are considered to be sensitive and valid to measure individual differences in inhibition. Accordingly, the main effects (See Results) were consistent with previous work [22–24], which indicates external reliability.

#### Go/No-go task

**Fig 3:**
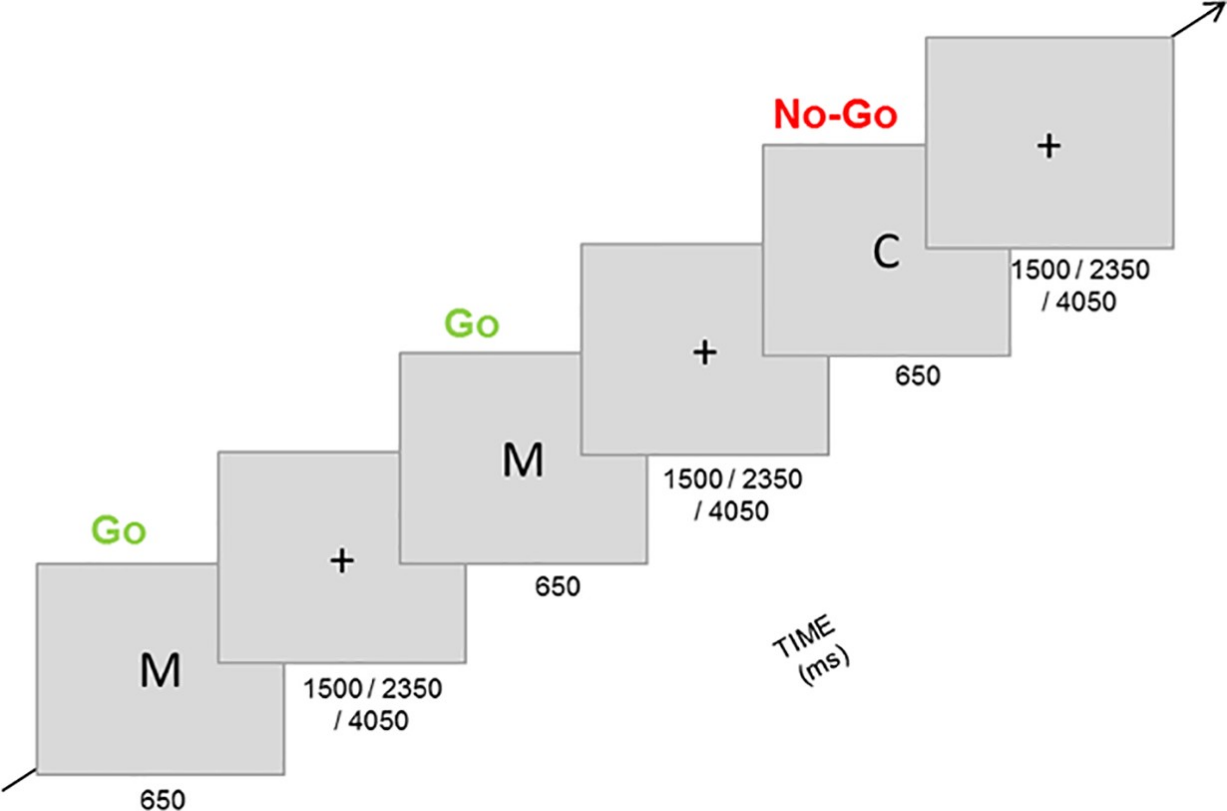
General inhibition—Go/No-go task. Example of a Go/No-go task trials sequence.

This paradigm was used to target *general inhibition*. Participants were instructed to respond to a frequent Go stimulus and to not respond to an infrequent No-go stimulus. They responded with the right index finger on a button-box (Fig 3). As stimuli, the letters ‘C’ and ‘M’ were used and which letter was defined as the Go or No-go stimulus was counterbalanced across participants. Participants had to complete four blocks of 80 trials each (25% No-go trials). Every trial consisted of the presentation of the stimulus for 200 ms, followed by an inter-trial interval of 1500, 2350, or 4050 ms (Fig 3). Hits (responding to a Go trial), correct No-go’s (not responding to a No-go trial), false alarms (responding to a No-go trial), and misses (not responding to a Go trial) were recorded. Responses after 650 ms with respect to stimulus onset were not registered. The letters (3 X 2.3 cm) were displayed in white color on a gray background [7, 20]. The three computer-based tasks were programmed and presented with PsychoPy [27].

Prior to entering the MR Scanner, participants performed a brief practice session for each task. There were twenty practice trials for the Approach-Avoidance Task (AAT), eight for the Negative Affective Priming task (NAP), and ten for the Go/No-go task. The practice trials involved different stimuli than the actual tasks (animals and plants for the AAT and NAP tasks, and ‘T’ and ‘K’ letters for the Go/No-go task).

### Technical details and fMRI acquisition

Participants performed all paradigms inside the MR Scanner. Data were acquired at the 3 T Siemens Prisma Scanner at the Maastricht Brain Imaging Center, Maastricht University. Functional EPI images were collected using an in-house developed multi-echo multi-band sequence (TR = 850 ms, TE = 15/30/44 ms, flip angle = 50°, FOV = 210 mm, 36 slices, isovoxel 3 mm^3^). Online-scanner reconstruction was performed using the slice-GRAPPA algorithm [28] with leakage artifact reduction [29] as implemented in the reconstruction of the MGH blipped-CAIPI SMS-EPI distribution (software and complete documentation are available at https://www.nmr.mgh.harvard.edu/software/c2p/sms). This GRAPPA sequence was selected to optimize the BOLD signal in frontoventral regions.

High-resolution anatomical images were acquired with a MPRAGE sequence (TR = 2250 ms, TE = 2.21 ms, FOV = 256 mm, 192 sagittal slices, isovoxel 1 mm^3^). The acquisition of anatomical images was done after the Go/No-go task (see above) to avoid cognitive fatigue.

### fMRI analyses

The imaging data were pre-processed and analyzed with Brain Voyager 21 (Brain Innovation, Maastricht, Netherlands).

Prior to the pre-processing, the echo images were combined using an optimized echo weighting method [30]. The images were motion-corrected (trilinear / sinc interpolation and aligned to the first functional volume acquired after the anatomical sequence) and corrected for slice timing skew using temporal sinc interpolation. A temporal high pass filter (3 cycles) was applied. Images were co-registered to the individual T1 weighted images and normalized to Talairach stereotaxic space. Volume time courses were spatially smoothed using a 6mm full width half maximum Gaussian kernel.

To analyze the activation pattern of every task an event-related approach was implemented using a GLM model and a random-effects group analysis. For the sexual tasks, we executed 2 x 2 ANOVA analyses with Stimuli (Sex vs Non-Sex) and Inhibitory/Non-Inhibitory (AAT: Approach vs Avoid; NAP: Priming vs No Priming) conditions as factors. Further basic contrast analyses were executed to specifically compare sexual inhibitory conditions against the non-sexual inhibitory conditions (AAT: Sex Avoid > Dance Avoid; NAP: Sex Priming > Non-Sex Priming) and the sexual non-inhibitory conditions against the non-sexual non-inhibitory conditions (AAT: Sex Approach > Dance Approach; NAP: Sex No Priming > Non-Sex No Priming). In the case of the Approach-Avoidance task, the resizing of the stimuli was the same in the conditions within each contrast (i.e. halving the stimuli size for avoid conditions and doubling it for approach conditions), therefore keeping the intensity of the stimuli constant. Motion correction parameters were included as confound variables in the GLM. In the case of the Go/No-go task, we calculated the No-go > Go contrast to identify regions active in motor response inhibition. For the three tasks, only correct trials were analyzed, excluding the trials where the participants did not respond or committed errors. The resulting maps were corrected for multiple comparisons by means of cluster threshold level estimation (1000 Monte Carlo stimulation iterations; [31]). Only clusters with a minimum size of 300 voxels are reported. The nomenclature of the cluster peak values was defined with the software tool Talairach Client [32–33].

Conjunction analyses were conducted to investigate the common neural substrates underlying the different inhibition processes. We looked at the conjunction of the three processes as compared to their respective control condition (Sex Avoid > Dance Avoid ^ Sex Priming > Non-Sex Priming ^ No-go > Hits) and of the paired combinations (i.e. Sex Avoid > Dance Avoid ^ Sex Priming > Non-Sex Priming; Sex Avoid > Dance Avoid ^ No-go > Hits; Sex Priming > Non-Sex Priming ^ No-go > Go). In addition we performed the conjunction analyses for the non-inhibitory conditions from the sexual tasks (Sex Approach > Dance Approach ^ Sex No Priming > Non-Sex No Priming).

## Results

### Behavioral data

#### Approach-Avoidance task

A two-way repeated measures ANOVA (Stimulus Type [Sex, Dance] X Response [Approach, Avoid]) revealed a significant effect of stimulus type (F_(1,21)_ = 12.11, p = .002, η^2^ = .35) and an interaction between Stimulus Type X Response (F_(1,21)_ = 10.84, p = .01, η^2^ = .37) but no main effect of Response (F_(1,21)_ = .07, p = .79, η^2^ = .002). Consistent with previous studies [22–24], participants reacted faster when responding to sexual stimuli versus neutral stimuli (Sex: M = 1172, SD = 342 ms; Dance: M = 1213, SD = 319 ms) and overall, reaction times were shorter when approaching sexual stimuli compared to approaching and avoiding neutral stimuli (Table 1). Participants committed on average 1.66 errors and .56 misses (FAILS: Sex Approach M = 1.09, SD = 3.07; Sex Avoid M = 1.48, SD = 2.33; Dance Approach M = 1.57, SD = 1.78; Dance Avoid M = 2.52, SD = 4.73; MISSES: Sex Approach M = .43, SD = 1.47; Sex Avoid M = .52, SD = .95; Dance Approach M = .65, SD = 1.19; Dance Avoid M = .65, SD = 1.41).

**Table 1:** Average reaction times of the sexual inhibition tasks conditions.

**Approach-Avoidance Task**		
	Sex $\bar{x}$ (SD)	Dance $\bar{x}$ (SD)
Approach	1150 (357)	1238 (326)
Avoid	1193 (331)	1187 (319)
**Negative Affective Priming Task**		
	Sex $\bar{x}$ (SD)	Non-Sex $\bar{x}$ (SD)
Priming	851 (147)	883 (160)
No Priming	828 (194)	857 (160)

#### Negative affective priming task

As expected, participants were slower to respond to Priming trials compared to No Priming trials (F_(1,21)_ = 4.9, p = .04, η^2^ = .16; Priming: M = 867, SD = 150; No Priming: M = 842, SD = 171 ms), and to non-sexual trials compared to sexual trials (F_(1,21)_ = 7.51, p = .01, η^2^ = .24; Sexual: M = 839, SD = 165 ms; Non-Sexual: M = 870, SD = 157 ms). No significant interaction was found between Stimulus Type and Priming condition (F_(1,21)_ = .02; p = .88, η^2^ = .001; Table 1). Participants committed on average .49 errors and .22 misses (FAILS: Sex Priming M = .74, SD = 1.13; Non-Sex Priming M = .52, SD = .79; Sex No Priming M = .35, SD = .64; Non-Sex No Priming M = .35, SD = .49; MISSES: Sex Priming M = .31, SD = .47; Non-Sex Priming M = .22, SD = .42; Sex No Priming M = .13, SD = .34; Non-Sex No Priming M = .22, SD = .42).

#### Go/No-go task

Participants committed on average 11 (SD = 14.46) misses and 7 (SD = 6.3) false alarms.

### fMRI results

#### Approach-Avoidance task

**Fig 4:**
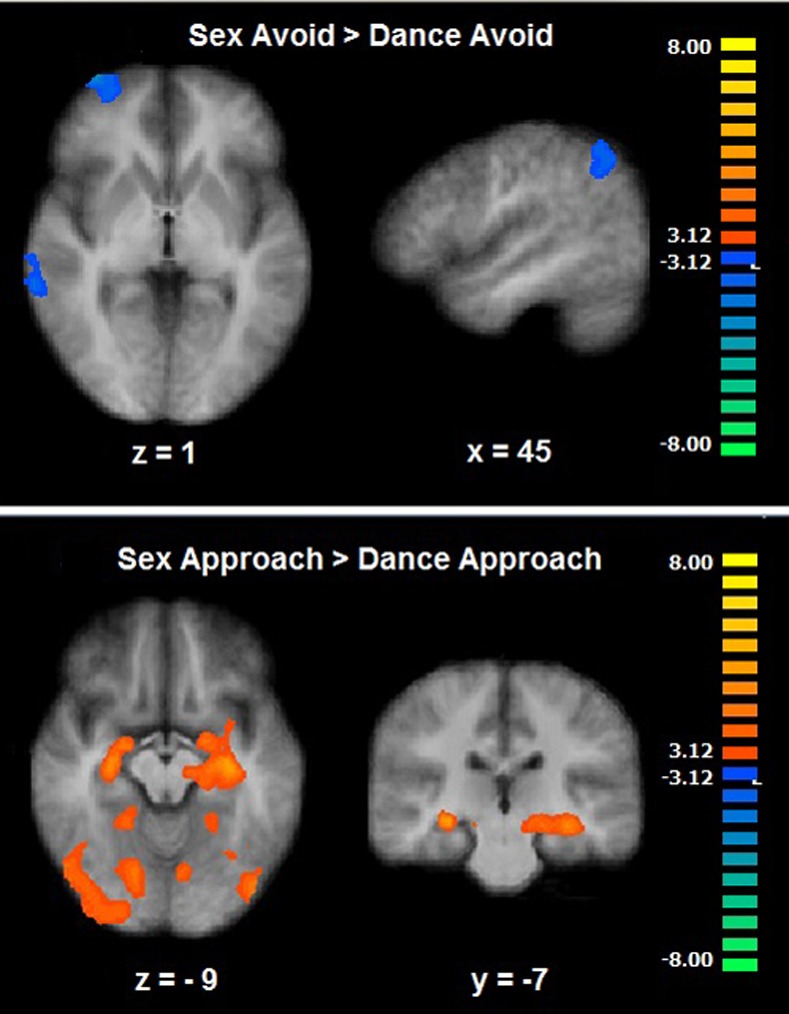
Motivational sexual inhibition—Approach-Avoidance task. Brain activation during the Sex Avoid (up) and Sex Approach trials (down) (CLTC, p < .005).

A two-way ANOVA (Stimulus Type [Sex, Dance] X Response [Approach, Avoid]) showed a significant Stimulus Type X Response interaction effect in the the parahippocampal gyrus, the cuneus (CLTC [cluster-level threshold correction], p < .001), and the lentiform nucleus extending to the amygdala (CLTC, p < .005; Table 2). When looking at the sexual avoidance condition map (Sex Avoid > Dance Avoid) a significantly decreased BOLD response was observed in the middle temporal gyrus, the inferior parietal lobule, the anterolateral prefrontal cortex, and the cuneus (CLTC p < .005; Table 2; Fig 4).

**Table 2:** Motivational sexual inhibition—Approach-Avoidance task.

	BA	x		y	z	Size (mm^3^)	F / t	P
*Stimuli x Movement Interaction*								
Parahippocampal Gyrus		-30		-16	-11	306	22.47	.00009
Cuneus	30	9		-67	7	568	19.76	.0002
Lentiform Nucleus**		18		-4	-5	441	20.18	.0001
*Sex Avoid >* *Dance Avoid*								
Middle Frontal Gyrus**	10	33		62	1	664	-5.05	.00005
Middle Temporal Gyrus**	21	63		-31	-2	1443	-4.55	.0001
Inferior Parietal Lobule**	40	45		-58	43	989	-4.41	.0002
Cuneus**	30	9		-67	7	579	-4.12	.0004
*Sex Approach > Dance Approach*								
Hippocampus		-30		-16	-11	1823	6.97	.000001
Parahippocampal Gyrus	28	27		-22	-5	1009	5.95	.000005
Middle Occipital Gyrus	18	39		-85	1	6578	5.61	.00001
Fusiform Gyrus	19	-42		-76	-11	938	5.19	.00003
Posterior Cingulate	30	6		-58	7	621	4.59	.00014
Cerebellum Posterior Lobe		-27		-89	-31	405	4.49	.00018

CLTC .001

** CLTC p < .005

**Fig 5:**
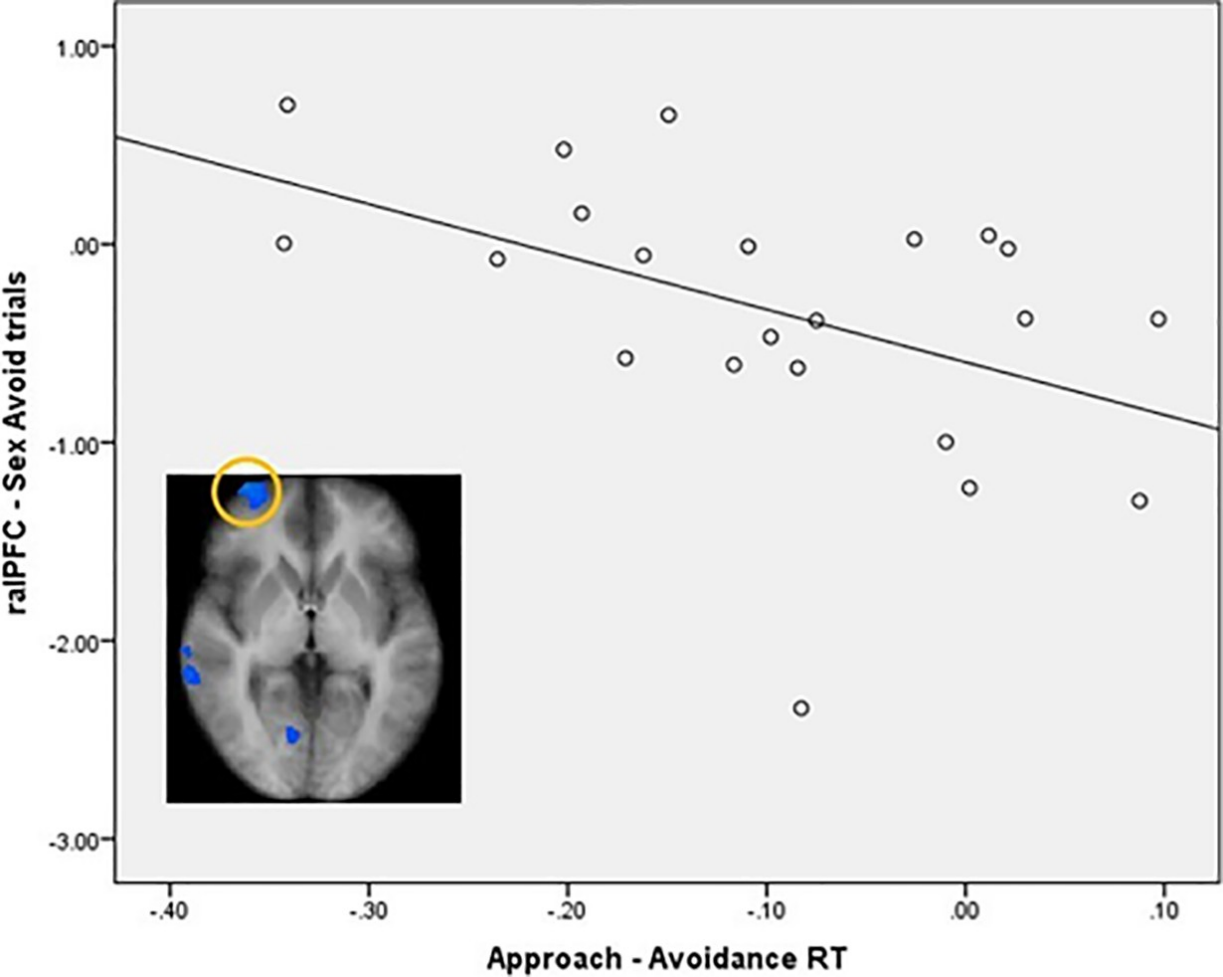
Brain activity correlates with the behavioral output in the Approach–Avoidance task. The activity of the right anterolateral prefrontal cortex during Sex Avoid trials negatively correlated with the Approach-Avoidance index (faster sexual avoidance and-or slower sexual approach reaction times).

When looking at the sexual approach condition map (Sex Approach > Dance Approach) an increased activation was observed in the middle occipital gyrus, the fusiform gyrus, the posterior cingulate, the cerebellum, the hippocampus, and the parahippocampal gyrus extending to the amygdala and to the lentiform nucleus (CLTC p < .001; Table 2; Fig 4). Because the anterolateral prefrontal cortex has been previously described as part of a self-regulation network [34] and in the current paradigm showed to be hypoactive during the sexual inhibitory condition we explored its relation with behavior. A correlation analysis showed a negative relation between the activity of this region (spherical ROI: x, y, z = 30, 59, 2; size: 257 voxels) and the main Approach-Avoidance reaction times index, indicating that participants who showed a stronger sexual inhibition (higher sexual avoidance together with lower sexual approach) showed a stronger anterolateral prefrontal cortex deactivation during the Sexual Avoid trials (r = -.46, p = .03; without outlier: r = -.58, p = .005, Fig 5).

#### Negative affective priming task

**Fig 6:**
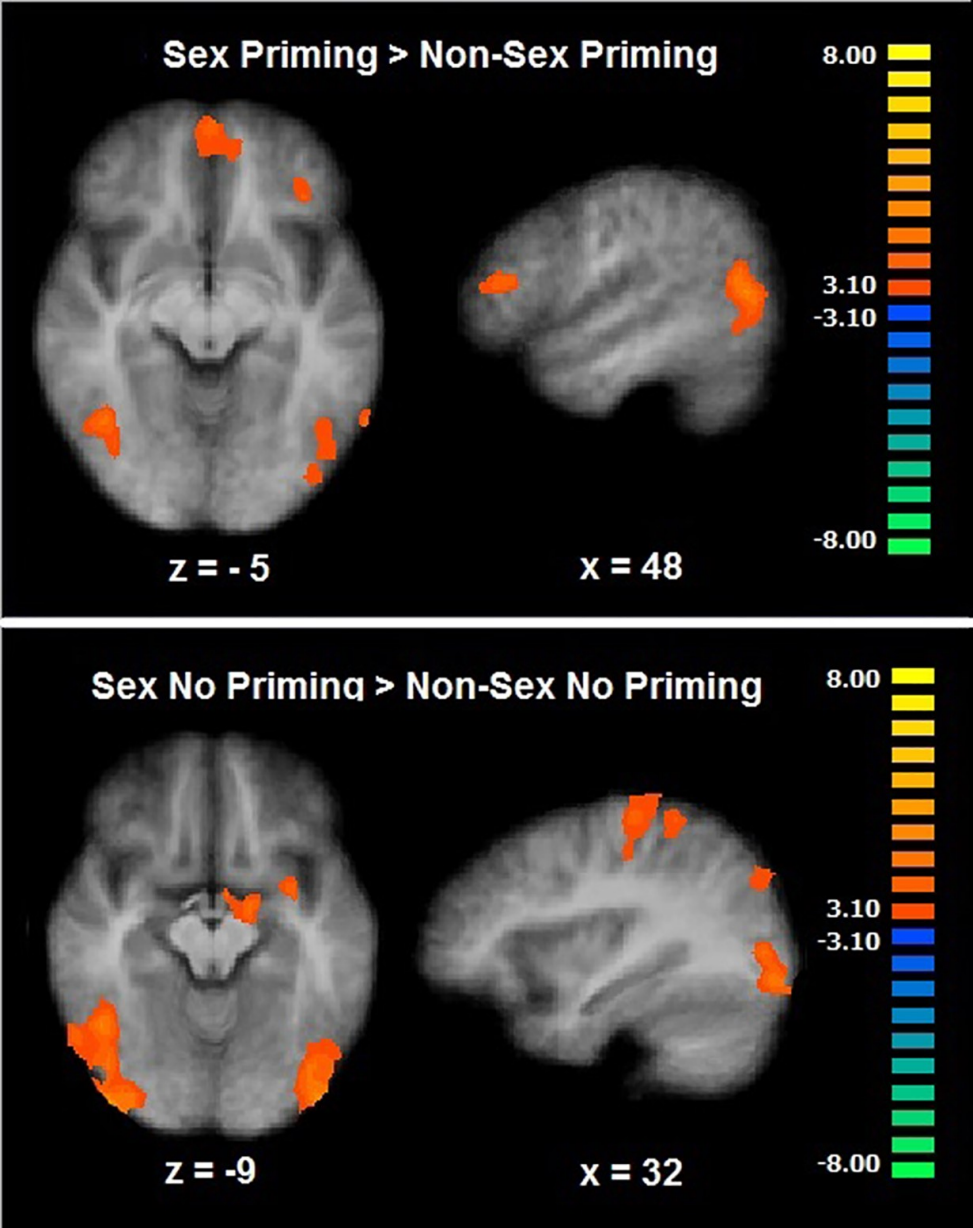
Cognitive sexual inhibition—Negative affective priming task. Brain activation during the Sex Priming (up) and Sex No Priming trials (down) (CLTC, p < .005).

A two-way ANOVA (Stimulus Type [Sex, Non-Sex] X Priming [Priming, No Priming]) showed a significant Stimulus Type X Priming interaction effect in the post-central gyrus (CLTC p < .001; Table 3). The sexual priming condition (Sex Priming > Non-Sex Priming) map revealed a significant increased BOLD response in the middle temporal gyrus, the posterior cingulate (CLTC p < .001), the inferior frontal gyrus, the middle frontal gyrus (orbitofrontal region), the medial prefrontal cortex, and the fusiform gyrus (CLTC p < .005; Table 3; Fig 6). The sexual No Priming condition map (Sex No Priming > Non-Sex No Priming) showed a significant enhanced activation in the cingulate gyrus, the post-central gyrus, the middle and superior temporal gyri, the inferior occipital gyrus, the medial prefrontal cortex, the parahippocampal gyrus (CLTC p < .001) and the lentiform nucleus (CLTC p < .005; Table 3; Fig 6). As the inferior frontal gyrus has been widely investigated in self-control and inhibition literature [35], we explored the relationship between its activation during the sexual priming trials and the sexual priming behavioral outcome (main NAP reaction times index). The relationship between the beta parameters in this region (spherical ROI: x, y, z = 46, 30, 13; size: 257 voxels) during the sexual priming condition and the NAP index was not significant (r = .21, p = .35).

**Table 3:** Cognitive sexual inhibition—Negative affective priming task.

	BA	x	y	z	Size (mm^3^)	F / t	P
*Stimuli x Priming Interaction*							
Post-central Gyrus	2	30	-34	61	547	14.58	.0008
*Sex Priming > Non-Sex Priming*							
Middle Temporal Gyrus	37	42	-64	10	2165	6.22	.000002
Posterior Cingulate	23	0	-52	22	738	5.08	.00003
Inferior Frontal Gyrus**	46	45	29	13	874	4.67	.0001
Fusiform Gyrus**	37	-45	-37	-11	561	4.41	.0002
Medial Frontal Gyrus**	10	0	56	-6	1455	3.21	.0003
Middle Frontal Gyrus**	11	-36	35	-9	866	4.05	.0004
*Sex No Priming > Non-Sex No Priming*							
Cingulate Gyrus	31	3	-40	37	3149	6.12	.000003
PostCentral Gyrus	2	-54	-25	37	757	5.41	.00001
Middle Temporal Gyrus	37	45	-61	-2	2808	5.34	.00002
Superior Temporal Gyrus	22	-48	-58	16	1455	5.31	.00002
Inferior Occipital Gyrus	18	-42	-83	-11	1319	5.27	.00002
Medial Frontal Gyrus	10	-9	47	1	3251	5.07	.00004
Parahippocampal Gyrus	34	18	2	-14	339	4.74	.00008
Lentiform Nucleus**		-15	-10	-8	1597	4.96	.0001

CLTC .001

** CLTC p < .005

#### Go/No-go task

**Fig 7:**
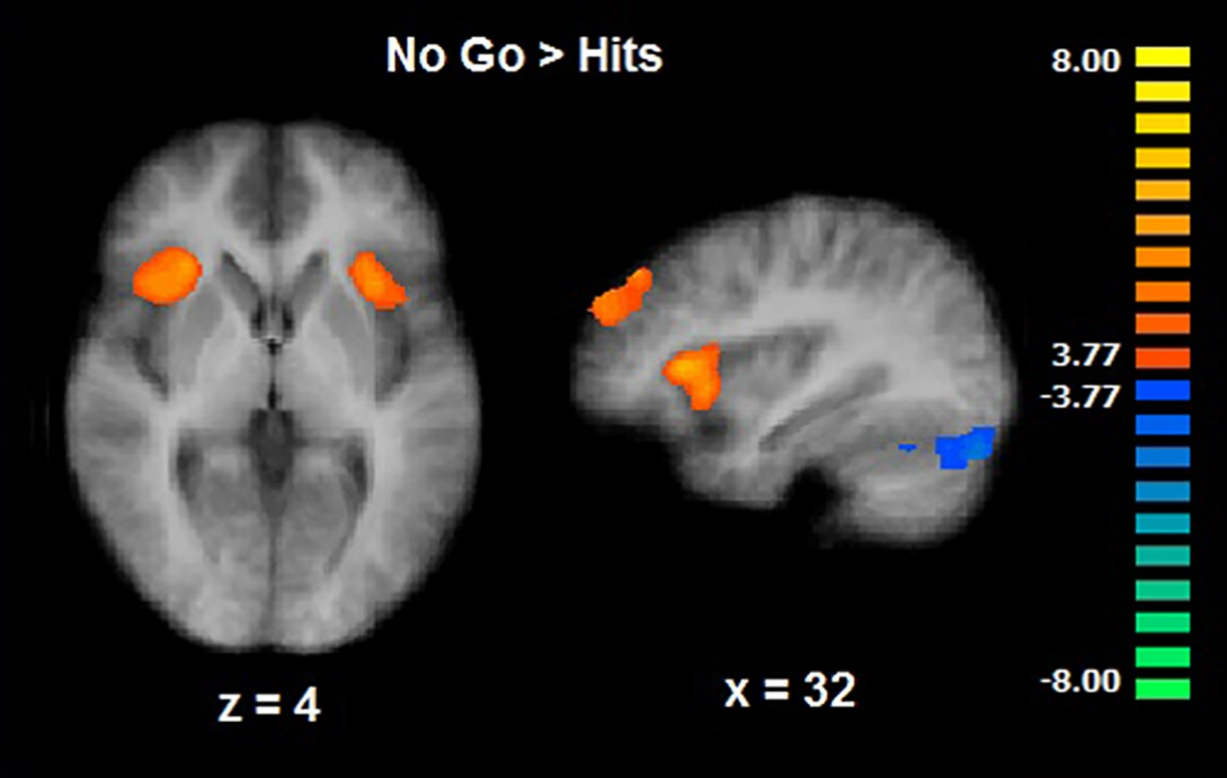
General inhibition–Go/No-go task. Brain activations during the No-go trials (CLTC p < .001).

When performing the No-go > Go contrast an increased activation in the right superior frontal gyrus, bilateral insula, extending to the inferior frontal gyrus on the right side, was observed (CLTC p < .001; Table 4; Fig 7). As we did with the motivational sexual inhibition task, we explored a relationship between the activity in the inferior frontal gyrus during the No-go condition (spherical ROI: x, y, z = 44, 18, 10; size: 257 voxels) and behavioral measures (number of false alarms and misses). This region of interest was created from the No-go > Go activation map. No correlation was significant (False Alarms: rho = .04, p = .85; Misses: rho = -.19, p = .35).

**Table 4:** General inhibition—Go/No-go task.

	BA	x	y	z	Size (mm^3^)	t	P
*No-go > Go*							
Claustrum		27	17	-2	5447	7.56	.0000001
Culmen		21	-46	-20	29815	-8.88	.0000001
Caudate Body		15	-10	25	3333	-7.77	.0000001
Middle Temporal Gyrus	39	-30	-55	22	4984	-7.16	.0000001
Postcentral Gyrus	3	-33	-31	55	8699	-7.21	.0000001
Insula	13	-33	20	13	2595	7.28	.0000001
Thalamus		0	-13	16	1823	-5.71	.000008
Substania Nigra		-15	-22	-8	607	-5.71	.000008
Postcentral Gyrus	43	-51	-19	16	2729	-6.64	.000001
Superior Frontal Gyrus	9	30	50	31	2023	5.84	.000006
Caudate Head		-3	11	4	2236	-6.01	.000004
Cuneus	18	3	-97	19	781	-4.79	.00007

CLTC p < .001

#### Conjunction analysis

The conjunction analyses of the three inhibitory processes contrasted against their respective control condition (Sex Avoid > Dance Avoid ^ Sex Priming > Non-Sex Priming ^ No-go > Go) did not reveal any overlapping region.

**Fig 8:**
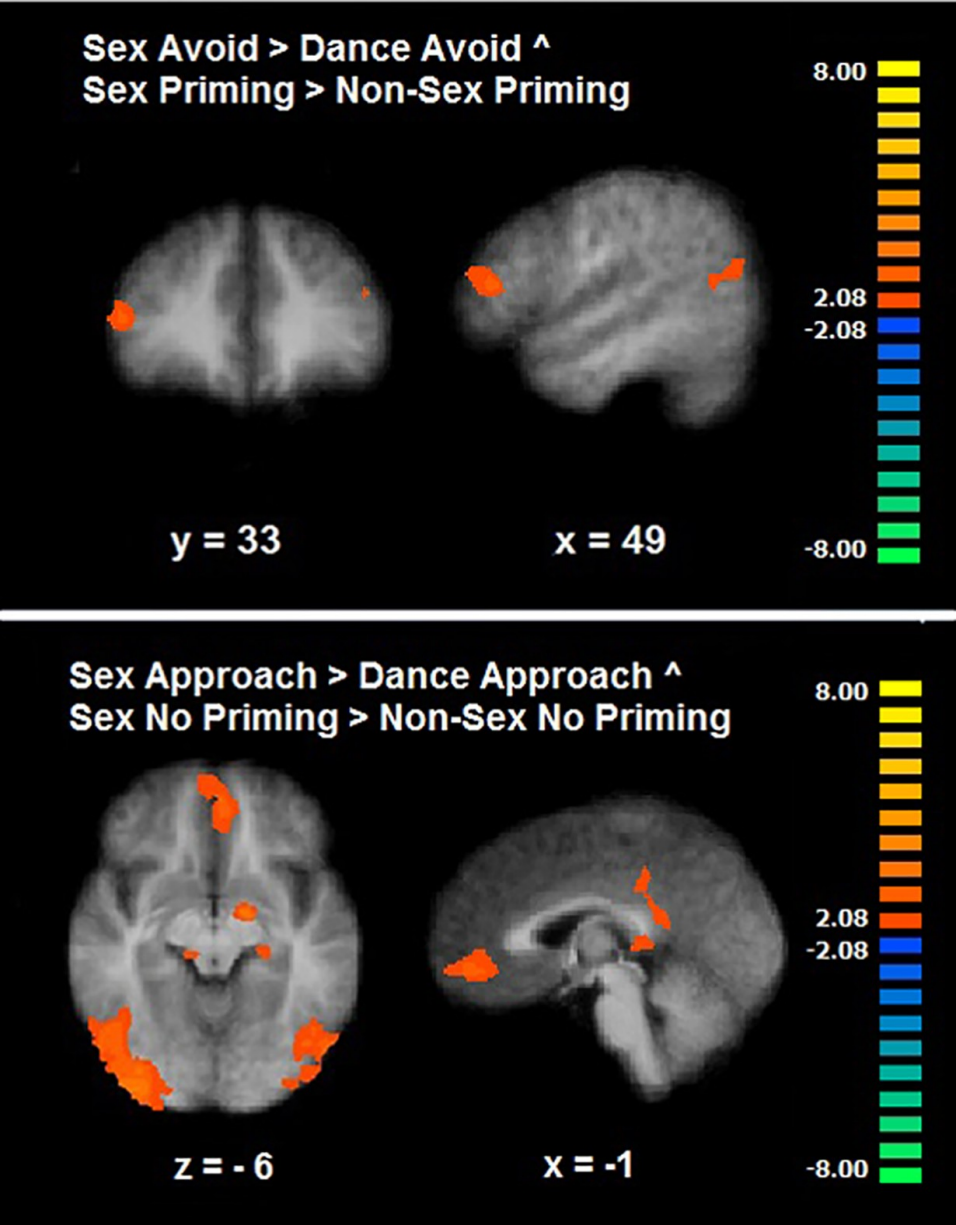
Conjunction of motivational and cognitive sexual inhibition. Common brain activation during the two sexual inhibitory conditions (up: Sex Avoid and Sex Priming) and during the two sexual non-inhibitory conditions (down: Sex Approach and Sex No Priming) (CLTC, p < .05).

For the two sexual inhibitory processes (Sex Avoid > Dance Avoid ^ Sex Priming > Non-Sex Priming), the analysis revealed an overlap in the inferior frontal gyrus and in the inferior and middle temporal gyri only at a liberal threshold of significance (CLTC p < .05). We also performed the conjunction of the two non-inhibitory sexual conditions from the two sexual tasks (Sex Approach > Dance Approach ^ Sex No Priming > Non-Sex No Priming). This analysis showed a common activation in the anterior and posterior cingulate, in the thalamus, the precuneus, the inferior occipital gyrus and the lentiform nucleus (CLTC p < .05; Table 5; Fig 8). Regarding the conjunction analyses of the Go/No-go task individually with each of the sexual tasks, results showed no overlapping even at a liberal threshold (CLTC p < .05).

**Table 5:** Conjunction analyses results for the three inhibitory processes and their paired combinations.

	BA	x	y	z	Size (mm^3^)	t	P
*Sex Avoid > Dance Avoid ^ Sex Priming > Non-Sex Priming ^ No-go > Go*							
	-	-	-	-	-	-	ns
*Sex Avoid > Dance Avoid ^ Sex Priming > Non-Sex Priming*							
Middle Temporal Gyrus	37	42	-58	-5	1330	3.51	.002
Inferior Temporal Gyrus	37	-45	-64	-2	3362	3.36	.002
Inferior Frontal Gyrus	46	48	29	13	905	3.18	.004
Middle Temporal Gyrus	39	48	-64	19	816	2.81	.01
*Sex Priming > Non-Sex Priming ^ No-go > Go*							
	-	-	-	-	-	-	ns
*Sex Avoid > Dance Avoid ^ No-go > Go*							
	-	-	-	-	-	-	ns
*Sex Approach > Dance Approach ^ Sex No Priming > Non-Sex No Priming*							
Inferior Occipital Gyrus	18	33	-88	-5	16319	4.36	.0002
Inferior Occipital Gyrus	18	-43	-82	-11	9887	4.17	.0004
Lentiform Nucleus		-15	-4	-8	984	4.19	.0004
Thalamus		-12	-31	4	3554	3.88	.0008
Posterior Cingulate	30	15	-52	13	2522	3.17	.004
Anterior Cingulate	32	-6	41	-5	2908	3.34	.003
Cingulate Gyrus	24	0	-10	34	588	3.24	.003
Parahippocampal Gyrus	35	18	-16	-8	325	2.77	.011
Precuneus	19	30	-79	37	1522	2.81	.011
Precuneus	7	21	-61	52	330	2.67	.014
Pyramis (cerebellum)	10	15	-64	-27	568	2.64	.015

CLTC p < .05.

## Discussion

In this study, we aimed at characterizing the neural correlates of sexual inhibition and at exploring whether there are common or distinct networks for cognitive versus motivational sexual inhibition, and how these networks relate to the networks recruited during general response inhibition. For this purpose, we used two different paradigms to target cognitive sexual inhibition (Negative Affective Priming) and motivational sexual inhibition (Approach-Avoidance), in addition to a classic Go/No-go paradigm to target general response inhibition. In a within-subject design, participants were required to execute all of these inhibition tasks while assessing their task-related whole-brain BOLD signal changes using fMRI.

To our knowledge, this is the first neuroimaging study that directly compares the neural networks underlying general inhibition and two types of sexual inhibition with a within-subject within-session design. The paradigms that we selected to target general inhibition, cognitive sexual inhibition and motivational sexual inhibition differed considerably in their design. This is important as it has been argued that the neural network associated with classic inhibitory paradigms reflects non-inhibitory processes inherent to the design (e.g. infrequent stimuli detection during No-go trials in the Go/No-go task). Therefore, by using different designs we reduced the risk of finding common neural mechanisms of non-inhibitory psychological processes associated with one particular paradigm.

Our findings demonstrate that whereas the motivational sexual inhibition is distinguished by a prefrontal hypoactivation pattern, cognitive sexual inhibition is characterized by activation in the ventromedial and inferolateral prefrontal regions. Nonetheless, both sexual inhibitory processes show a common activation in the inferior frontal gyrus and in the inferotemporal cortex. The general inhibition paradigm engaged the insula and the right inferior frontal gyrus, which is in accordance with previous literature [7, 20]. We will now discuss the activation pattern of every sexual inhibitory process followed by a discussion of the different inhibitory commonalities.

### Motivational sexual inhibition

The inhibitory control to avoid sexual stimuli was characterized by a hypoactivation in the anterolateral prefrontal cortex, the inferior parietal lobe, the middle temporal gyrus, and the cuneus. Of particular relevance is the observed hypoactivation of the anterolateral prefrontal cortex. This region was previously engaged in a similar approach-avoidance paradigm, being active in incongruent conditions (approaching angry faces and avoiding happy faces) as compared to congruent conditions [34]. It is intriguing that in the current study this region was hypoactive during the self-regulation (avoid sexual stimuli) condition.

It is possible that in our paradigm, a tonic inhibitory process was induced by the mere exposure of sexual pictures during the tasks, which was ‘released’ when participants deliberately aimed at avoiding the sexual images. Remarkably, the activation in this region correlated with the Approach-Avoidance main index. Individuals with a stronger inhibition of sexual stimuli (by avoiding them faster and/or taking longer to approach them) showed a stronger hypoactivation in the anterolateral prefrontal cortex during the sex avoiding trials. If it was the case that the anterolateral prefrontal cortex is sustaining a tonic inhibitory process, this would indeed explain why this mechanism was more active in individuals with a stronger motivational inhibition of sexual stimuli.

In contrast to the inhibitory control condition, approaching sexual stimuli largely engaged subcortical areas (the lentiform nucleus and the amygdala) that have been associated with sexual arousal and penile tumescence [16]. Lesions in the amygdala have led to hypersexuality in some patients [15]. Similarly, an irregular functional activation has been found in the lentiform nucleus of sexual compulsive patients [36]. This seems to indicate that not only the integrity of inhibitory networks is relevant for successful control of sexual behavior, but also the integrity of regions engaged in sexual approach. This is in concordance with the dual control model of male response [37], which states that the balance between sexual excitatory and sexual inhibitory mechanisms is essential for the regulation of sexual behavior and a disproportional high sexual excitation or disproportional low sexual inhibition can lead to hypersexual behavior.

### Cognitive sexual inhibition

The Negative Affective Priming paradigm allowed us to target the neural correlates of inhibiting sexual information at a cognitive level. The left orbitofrontal cortex, the fusiform gyrus, the middle temporal gyrus, the posterior cingulate, and the right inferior frontal gyrus were engaged during cognitive sexual inhibition. Although an inhibitory role has often been ascribed to the right inferior frontal gyrus and generally to the ventrolateral prefrontal cortex [4], previous work has shown that the temporal disruption of this region with transcranial magnetic stimulation led to an increase in cognitive sexual inhibition after accounting for sexual excitation scores [38]. Recent evidence shows that the inferior frontal gyrus is sensitive to processes inherent to inhibition paradigms such as target detection, and therefore not exclusive to inhibition itself [39]. Moreover, in the current study, the activation level of this region did not correlate with the behavioral inhibitory index. Therefore, the inferior frontal gyrus seems to play a relevant role during cognitive sexual inhibition but it does not seem to code for sexual inhibition itself.

The left orbitofrontal cortex has been proposed to exert an inhibitory tonic control over sexual stimuli, as it has shown to be hypoactive during different sexual cognition paradigms. In addition, this proposed inhibitory mechanism is dependent on testosterone levels, as this pattern (orbitofrontal deactivation towards sexual stimuli) is not observed in hypogonadal men and it is restored after testosterone administration [15, 40]. In addition, the lesion to the orbitofrontal cortex often leads to impairments in socio-affective regulation including sexual inhibition [15]. Its activation during the current study provides direct evidence for its engagement during the inhibition of incoming sexual stimuli (cognitive sexual inhibition). Because this inhibitory process occurs without the awareness of the individual (is not deliberate), it may actually constitute a default, or tonic, inhibitory mechanism.

Finally, we found that the fusiform gyrus was also active during cognitive sexual inhibition. The inferotemporal cortex, where the fusiform gyrus is located, has been suggested to act as a tonic inhibitor in the control of sexual behavior, as its lesion or resection has led to hypersexuality [15, 41]. However, it is unclear whether the fusiform gyrus was compromised in the brain damage. Although the function typically attributed to the fusiform gyrus is the recognition of complex visual patterns [42], there is growing evidence showing the involvement of the fusiform gyrus in inhibition, emotion regulation (e.g. [7, 19, 43–46]) and in non-visual sexual cognition [47, 48]. Thus, it could also be possible that its engagement during this paradigm represents an adaptive signaling during the inhibitory process through pattern recognition that is functionally guided (e.g. through an increased attention in faces).

### Overlap between motivational sexual and cognitive sexual inhibition

We posed the question whether inhibiting sexually driven motor actions and inhibiting sexually incoming information would share common neural substrates. The motivational sexual and cognitive sexual inhibitory conditions showed an overlap in the inferior frontal gyrus and in the inferior and middle temporal gyri. As was discussed before, although frequently associated with response inhibition [49], the exclusively inhibitory role of the inferior frontal gyrus has been challenged and recent evidence suggests a role in detecting sexual salient cues during cognitive sexual inhibition [38]. The fact that this area was commonly active during the two sexual inhibitory but not in the sexual non-inhibitory conditions, suggests that this region is not only sensitive to detecting salient sexual stimuli but in detecting them in function of other cognitive demands, which is inhibition in this case.

The inferior and medial temporal gyri were also conjointly active during the two types of sexual inhibition. The posterior inferotemporal cortex has found to be active during the perception of sexual stimuli and to vary according to the levels of sexual arousal and penile tumescence [50–51]. It is noteworthy that in the current study the conjoint activation of the posterior inferotemporal cortex appeared in the sexual inhibitory conditions (cognitive sexual and motivational sexual inhibition) but not in the sexual non-inhibitory conditions. This observation seems to support the notion that posterior inferotemporal regions play a specific role during sexual inhibition. Similar to the role of the fusiform gyrus–also part of the posterior inferotemporal cortex- during cognitive sexual inhibition, the posterior inferior and medial temporal gyri can play a part in recognizing patterns that are adaptively relevant during the inhibitory processes.

The pattern of activation during the two sexual inhibitory conditions largely contrasts with the pattern of the sexual non-inhibitory conditions from the two tasks. The latter revealed a common activation in the anteromedial prefrontal cortex which is associated with the subjective experience of sexual arousal [16]. In addition, there was a conjoint activation in the globus pallidus which when lesioned has led to hypersexuality in some clinical cases [15]. Research has shown that the structure and functionality of other basal ganglia regions are related to the frequency of sexual behavior and shows an abnormal pattern in hypersexual individuals [36, 52]. The neural activation pattern of sexual inhibition in this study did not reveal any activation in the basal ganglia. This shows once again, that the integrity of both, sexual excitatory and inhibitory networks, is important for the successful control of sexual manifestations.

### Overlap between sexual and general inhibition

Given the coexistence of sexual and general inhibition deficits in different clinical conditions, we also investigated whether sexual inhibition shares common neural mechanisms with general inhibition. We did not find commonalities among cognitive sexual inhibition, motivational sexual inhibition, and general inhibition. The absence of common neural networks in sexual and general inhibition seems to contrast with clinical observations showing that frontal lobe damaged patients are unable to control both their sexual and non-sexual behavior. Different explanations may account for this finding: 1) The three inhibitory processes recruited different but adjacent portions of the ventrolateral prefrontal cortex, therefore an extensive lesion in the area would lead to a generalized impairment in different inhibitory modalities. 2) The Go/No-go paradigm lacks a socio-affective component; the general inhibition impairment reported in frontal lobe lesion patients often refers to a disregard for social or even moral norms [53–54]. Thus, the link between general and sexual inhibition may rely on processes involving social and/or affective cognition. 3) Although the Go/No-go task does not relate to other sexual inhibitory processes *per se*, general inhibition may influence non-sexual processes that ultimately relate to sexual behavior; for instance, a lack of inhibition in social contexts could lead to more sexual encounters.

Another potential contradiction to our findings is that sexual compulsion is often comorbid with other disinhibition related behaviors such as substance abuse. Even if sexual and general inhibition recruited different neural substrates, some conditions could influence different networks indistinctively. For example, a deficit in dopamine would have an effect on cognitive, motor, and motivational circuits.

### Limitations and future directions

While our study allowed the characterization of the neural circuits sustaining sexual inhibitory mechanisms, one should consider that a possible limitation of the study is that only male participants were tested. Since women are less vulnerable to sexual inhibition impairments such as in hypersexuality [55], and women and men show important differences in sexual cognitive processes [23] and inhibitory ones [56–57], we included only men in our sample to provide a first test of the role of inhibition in sexual responding. However, future studies should also target sexual inhibitory processes in women and see whether these differ from patterns observed in men.

Regarding the technical part of the method, future studies can benefit from two particular points in the acquisition and in the analysis of the data. First, future studies may benefit, as we did, from the use of fMRI sequences that optimizes the signal in frontoventral regions. These regions are generally susceptible to noise artifacts, but are nevertheless crucial in socio-affective processing. Second, although our design provided the spatial accuracy and functional specificity that was not given by other methods or paradigms, a better comprehension of the neural circuits sustaining sexual inhibition can be achieved by studying the network interactions through advanced methods such as dynamic causal modelling.

Finally, it is noteworthy that the regions found to be common in the inhibitory and non-inhibitory conditions of the sexual tasks were only observed at a very liberal threshold of significance (p = .05, CLTC) which may lead to false positives. Therefore, these results and its interpretation should be considered with caution.

In sum, this study did not support the existence of common general and sexual inhibitory networks. However, the inhibition of a sexually motivated driven action and the attentional inhibition of sexual information commonly engaged the inferior frontal gyrus and the posterior inferotemporal cortex. The specific functional properties of these regions, as well as those of the individual networks (for cognitive sexual and motivational sexual inhibition) remain to be studied in order to understand the distinct symptomatology and comorbidity of sexual disorders.
